# COVID-19 as ‘Game Changer’ for the Physical Activity and Mental Well-Being of Augmented Reality Game Players During the Pandemic: Mixed Methods Survey Study

**DOI:** 10.2196/25117

**Published:** 2020-12-22

**Authors:** Louise A Ellis, Matthew D Lee, Kiran Ijaz, James Smith, Jeffrey Braithwaite, Kathleen Yin

**Affiliations:** 1 Centre for Healthcare Resilience and Implementation Science Australian Institute of Health Innovation Macquarie University North Ryde Australia; 2 NHMRC Partnership Centre in Health System Sustainability Australian Institute of Health Innovation Macquarie University North Ryde Australia; 3 School of Nursing University of Pennsylvania Philadelphia, PA United States; 4 Centre for Health Informatics Australian Institute of Health Innovation Macquarie University North Ryde Australia

**Keywords:** COVID-19, Pokémon GO, Harry Potter: Wizards Unite, augmented reality games, physical activity, mental health, well-being

## Abstract

**Background:**

Location-based augmented reality (AR) games, such as Pokémon GO and Harry Potter: Wizards Unite, have been shown to have a beneficial impact on the physical activity, social connectedness, and mental health of their players. In March 2020, global social distancing measures related to the COVID-19 pandemic prompted the AR games developer Niantic Inc to implement several changes to ensure continued player engagement with Pokémon GO and Harry Potter: Wizards Unite. We sought to examine how the physical and mental well-being of players of these games were affected during the unprecedented COVID-19 restriction period as well as how their video game engagement was affected.

**Objective:**

The aims of this study were to examine the impact of COVID-19–related social restrictions on the physical and mental well-being of AR game players; to examine the impact of COVID-19–related social restrictions on the use of video games and motivations for their use; and to explore the potential role of AR games (and video games in general) in supporting well-being during COVID-19–related social restrictions.

**Methods:**

A mixed methods web-based self-reported survey was conducted in May 2020, during which COVID-19–related social restrictions were enforced in many countries. Participants were recruited on the web via four subreddits dedicated to Pokémon GO or Harry Potter: Wizards Unite. Data collected included quantitative data on demographics, time spent playing video games, physical activity, and mental health; qualitative data included motivations to play and the impact of video games on mental health during COVID-19 lockdown.

**Results:**

We report results for 2004 participants (1153/1960 male, 58.8%, average age 30.5 years). Self-reported physical activity during COVID-19–related social restrictions significantly decreased from 7.50 hours per week on average (SD 11.12) to 6.50 hours (SD 7.81) (*P*<.001). More than half of the participants reported poor mental health (925/1766, 52.4%; raw World Health Organization–5 Well-Being Index score <13). Female gender, younger age, and reduced exercise were significant predictors of poor mental health. Participants reported a significant increase in video game play time from 16.38 hours per week on average (SD 19.12) to 20.82 hours (SD 17.49) (*P*<.001). Approximately three quarters of the participants (n=1102/1427, 77.2%) reported that playing video games had been beneficial to their mental health. The changes made to Pokémon GO and Harry Potter: Wizards Unite were very well received by players, and the players continued to use these games while exercising and to maintain social connection. In addition to seeking an escape during the pandemic and as a form of entertainment, participants reported that they used video games for emotional coping and to lower stress, relax, and alleviate mental health conditions.

**Conclusions:**

AR games have the potential to promote physical and mental health during the COVID-19 pandemic. Used by populations under isolation and distress, these games can improve physical and mental health by providing virtual socialization, sustained exercise, temporal routine, and mental structure. Further research is needed to explore the potential of AR games as digital behavioral interventions to maintain human well-being in the wider population.

## Introduction

COVID-19 was first reported in Wuhan, China, on December 31, 2019 [1,2], and it has since escalated to a global pandemic. COVID-19 is transmitted between humans in close proximity; therefore, physical (or social) distancing is a key measure for reducing its spread [3]. By April 2020, most countries worldwide had introduced quarantine measures and travel bans, cancelled social events, and closed public services to contain COVID-19 [4]. With the introduction of stay-at-home lockdown and quarantine measures, electronic video game playing reached an all-time high [5]. Reputedly, 82% of global consumers played video games and watched video game content during the height of the lockdown period during the COVID-19 pandemic [5]. Increased web-based gaming was viewed as complementary to public efforts to promote physical distancing [6]. Most notably, the World Health Organization (WHO) partnered with the gaming industry in March 2020 to launch the campaign #PlayApartTogether to encourage people to stay at home, play video games, and practice physical distancing [7].

Although web-based gaming has played a supporting role during the COVID-19 pandemic in maintaining physical distancing, concerns have been raised about encouraging video gaming [6]; previous research has linked excessive gaming with poor mental health, sleep problems, and physical inactivity [8]. Protracted periods of isolation, technology-based activity, and limited social interaction can also solidify unhealthy lifestyle patterns, intensify technology-related disorders, and potentially lead to difficulties of readaptation after the COVID-19 pandemic has ended [6].

For this reason, “healthier” gaming options that promote increased physical activity and social connection [6,9,10] are being encouraged. Location-based augmented reality (AR) games developed by Niantic Inc, such as Pokémon GO and Harry Potter: Wizards Unite [11], are of particular interest because they are designed to increase physical activity [12,13] and have also been found to increase players’ social connectedness [14] and mental well-being [12,15]. These games require players to explore outdoor public spaces and engage in social events as part of the normal play experience, using cellular, Wi-Fi, and GPS networks to determine the player’s approximate location. This in turn affects the player’s ability to interact with game features and other players.

With government-issued restrictions during the COVID-19 pandemic encouraging—or requiring—individuals to stay at home, playing AR games in the manner in which they were originally designed has become challenging [10,16]. Niantic Inc implemented several in-game changes to Pokémon GO and Harry Potter: Wizards Unite to prevent these restrictions from having deleterious effects on the player’s experience; therefore, these games are easier to play at home and in social isolation [17]. These changes ensured that Pokémon GO and Harry Potter: Wizards Unite continued to be popular [16]. However, these changes did not necessarily translate to improved physical and mental well-being for players, as many gameplay features designed to promote physical activity and social activity were removed or altered [16]. This study sought to examine the physical and mental well-being of players of Pokémon GO and Harry Potter: Wizards Unite during the COVID-19 pandemic as well as how players viewed the roles played by AR games and video games in general in their mental health during the pandemic.

As such, our aims were (1) to examine the impact of COVID-19–related social restrictions on the physical and mental well-being of AR game players; (2) to examine the impact of COVID-19–related social restrictions on use of video games and motivations for their use; and (3) to explore the potential role of AR games (and video games in general) in supporting players’ well-being during COVID-19–related social restrictions.

## Methods

### Overview

We conducted a mixed methods web-based survey during a period in which many countries were under social restrictions due to COVID-19 (May 15-29, 2020, inclusive) [4]. This survey is part of a larger study examining Pokémon GO and Harry Potter: Wizards Unite use and player experience during the COVID-19 pandemic. Ethical approval was obtained from the Human Research Ethics Committee for Medical Sciences of Macquarie University (Reference No: 52019601512435).

### Sample and Recruitment

Participant recruitment was conducted on the web via four subreddits dedicated to Pokémon GO or Harry Potter: Wizards Unite (r/WizardsUnite, r/PokemonGO, r/harrypotterwu, and r/TheSilphRoad). A recruitment post was pinned to the top of each of subreddit from May 15-29, 2020, directing individuals to the participant information page via Qualtrics [18]. Participants were informed that participation was voluntary, that they could withdraw at any time prior to submitting their final responses, and that to be eligible, they needed to be ≥18 years of age and have played the English versions of Pokémon GO or Harry Potter: Wizards Unite for at least a week. Those who consented to participate were directed to the web-based survey, which took 15 to 20 minutes to complete. No incentives were offered for taking part in the survey. Participants were informed not to provide any identifiable personal information. The IP addresses of the participants were recorded by Qualtrics to identify repeat visits. Duplicate entries were not permitted after a participant had submitted the survey.

### The Survey

The survey included a total of 40 questions, both quantitative and qualitative. Demographic information was collected from the participants, including age, gender, and country of residence. Responses from quantitative questions relating to video game use, exercise, and mental well-being are reported here, as well as responses from two qualitative questions regarding motivation to play and the impact of games on mental health (Multimedia Appendix 1).

#### Video Game Use

Participants were asked whether they identified as a “hardcore gamer,” a “casual gamer,” or a “midcore gamer” to identify their usual play patterns and levels of engagement [19]. Hardcore gamers were defined as those who invest a significant amount of their recreational time and resources into gaming and who also have more knowledge and skills related to games [19-21]. On the other hand, casual gamers were defined as those who play games casually, in short sessions, or infrequently [22,23]. Midcore gamers fall in between these categories and include people who regularly play video games but are not “super serious” gamers [23]. Participants were also asked for their typical video game playing frequency prior to the COVID-19 pandemic (number of hours per day and days per week) and during COVID-19 lockdown (number of hours per day and days per week). In addition, participants were asked whether they continued to play either Pokémon GO or Harry Potter: Wizards Unite during the lockdown (Yes/No).

#### Physical Activity

Respondents were asked to report their typical exercise frequency prior to the COVID-19 pandemic (number of hours per day and days per week) and during COVID-19 lockdown (number of hours per day and days per week).

#### Mental Well-being

The World Health Organization–5 Well-Being Index (WHO-5) [24] is a 5-item measure of current mental well-being. The WHO-5 consists of five items assessing positive mood, vitality, and general interest over the past two weeks, scored on a 6-point Likert scale from 0 (at no time) to 5 (all of the time). Scores are summed to create a total “raw score” (range 0-25), with lower scores indicating impaired emotional well-being [24]. A total “percentage score” is obtained by multiplying the total raw score by four. A raw score <13 (or <50%, or a score of 0 or 1 on any of the items) is considered to be an indicator of impaired mental well-being and of likely depression [25,26].

#### Qualitative Questions

This paper focuses on responses to two qualitative questions: (1) “What motivates you to play Pokémon GO or Harry Potter: Wizards Unite during the COVID-19 shutdown?” and (2) “How has playing video games affected your mental health during the shutdown?”

### Data Analysis

Quantitative survey data were analyzed using SPSS 25.0 (IBM Corporation). Reported total hours of participation in gaming and exercise per week were calculated by multiplying the number of hours by the number of days they were performed. Differences between male and female participants and between self-identified player types were examined using chi-square analysis or multinomial logistic regression for categorical dependent variables or analysis of variance (ANOVA) for continuous dependent variables. Repeated-measures ANOVA was also used to assess mean differences in hours of participation in gaming and exercise per week by gender and player type over time (ie, pre–COVID-19 and during COVID-19 lockdown). Finally, binary logistic regression was used to determine factors associated with impaired mental well-being. Here, game play and exercise change scores (pre–COVID-19 minus during COVID-19 lockdown) were incorporated in the analysis. Due to the large sample size, the significance level was set at *P*<.01 for all analyses.

Qualitative responses were analyzed via thematic analysis using NVivo (QSR International) [27]. Thematic analysis using a 6-step process [28] was undertaken independently by two researchers (LAE and MDL). Codes were developed using an iterative process to finalize and map important themes and ensure consistency with the data [29]. The broader research team were included throughout each stage of the analysis process to help resolve any differences, with frequent discussions concerning themes. The qualitative findings were used to triangulate and build upon the quantitative data.

## Results

### Demographic Results

In total, 2165 participants responded to the survey. The sample was reduced to 2004 after excluding participants with more than 30% missing survey data. In the sample, most participants were male (1153/1960, 58.8%) and aged between 25 and 34 years (n=985/1998, 49.3%, M=30.5, range:18-99). Participants were drawn from 66 different countries, although almost half were from the United States (987/1987, 49.7%). The characteristics of the survey respondents are presented in Table 1. Only countries with more than 50 respondents are shown.

**Table 1:** Demographic characteristics of survey respondents (N=2004), n (%). Values may not equal total N due to missing demographic responses.

Characteristic		Value
**Gender**		
	Female	807 (41.2)
	Male	1153 (58.8)
**Age (years)**		
	18-24	523 (26.2)
	25-34	985 (49.3)
	35-44	329 (16.5)
	≥45	161 (8.1)
**Country**		
	Australia	77 (3.9)
	Canada	146 (7.5)
	Germany	90 (4.5)
	The Netherlands	52 (2.6)
	United Kingdom	177 (8.9)
	United States	987 (49.7)
	Other	458 (23.1)
**Self-identified player type**		
	Casual gamer	905 (46.6)
	Hardcore gamer	392 (20.2)
	Midcore gamer	646 (33.2)

### Self-Identified Player Type

When asked to self-identify as a hardcore, casual, or midcore gamer, most respondents considered themselves casual gamers (905/1943, 46.6%), followed by midcore gamers (646/1943, 33.2%), and hardcore gamers (392/1943, 20.2%). Hardcore gamers were significantly more likely to be male (324/385, 84.2%) than female (61/385, 16.0%) (χ^2^_1901_=160.23, *P*<.001), and significantly younger than casual gamers (χ^2^_1293_=53.97, *P*<.001).

### Quantitative Results

#### Impact of the COVID-19 Pandemic on Physical Activity

Prior to the COVID-19 pandemic, participants reported exercising for an average of 7.50 hours per week (SD 11.12). At this time, hardcore players (mean 9.07 hours, SD 12.92) exercised significantly more than casual gamers (mean 7.19 hours, SD 11.10; *F*_1,1129_=6.14, *P*=.01), and men (mean 8.31 hours, SD 13.00) exercised significantly more than women (mean 6.35 hours, SD 7.88; *F*_1,1708_=12.88, *P*<.001). During the COVID-19 lockdown, participants decreased their exercise to an average of 6.50 hours per week (SD 7.81). Repeated measures ANOVA showed that this reduction in exercise from before to during the lockdown was significant (*F*_1,1058_=15.79, *P*<.001). A significant player type–by-time interaction was also identified, with hardcore gamers exhibiting a greater reduction in exercise during the COVID-19 lockdown than casual gamers (*F*_1,1058_=6.58, *P*=.01). There was no significant gender-by-time interaction. We also identified a significant positive correlation between total hours of gaming and exercise per week both during COVID-19 lockdown (*r*=0.198, *P*<.001) and prior to lockdown (*r*=0.257, *P*<.001).

#### Mental Well-being During COVID-19 Lockdown

Over half of the participants reported poor mental health (925/1766, 52.4%; raw WHO-5 score <13) at the time the survey was conducted. A multivariate binary logistic regression analysis was conducted with age, gender, self-identified gaming type, difference in video game play, and difference in exercise entered as predictors. The model identified being female (*P*<.001), being younger (*P*<.001), and reduced exercise (*P*=.008) as significant predictors of poor mental health during the COVID-19 lockdown. At a significance level of *P*<.01, self-identified player type (*P*=.22) and difference in video game play (*P*=.04) were not significant predictors of poor mental health (Multimedia Appendix 2).

#### Impact of COVID-19 Lockdown on Video Game Use

Three in four participants (1261/1675, 75.3%) reported that during the COVID-19 lockdown, they played video games “a little more” to “a lot more.” Of these 1675 participants, 20% (n=335) reported “no change,” and only 4.7% (n=79) reported playing “a little less” to “a lot less.” Prior to the COVID-19 pandemic, participants played video games for an average of 16.38 hours per week (SD 19.12). At this time, hardcore gamers (mean 20.24 hours, SD 22.47) played significantly more than casual gamers (mean 13.97, SD 17.32; *F*_1,1276_=29.27, *P*<.001), and men (mean 17.90, SD 20.77) played significantly more than women (mean 14.26, SD 16.34; *F*_1,1929_=17.03, *P*<.001). During the COVID-19 lockdown, participants increased their video game playing time to an average of 20.82 hours per week (SD 17.49). Repeated measures ANOVA indicated that the difference in video game playing time between the pre-lockdown and lockdown periods was significant (*F*_1,1260_=55.12, *P*<.001). Notably, a significant player type–by-time interaction was found; casual gamers increased their video game playing time during COVID-19 lockdown (mean 20.00 hours, SD 15.35) to almost match that of hardcore gamers (mean 21.84 hours, SD 19.91; *F*_1,1260_=16.83, *P*<.001). Similarly, a significant gender-by-time interaction was identified; women increased their video game playing time during COVID-19 lockdown (mean 20.91 hours, SD 17.43) to equal that of men (mean 20.82 hours, SD 17.71; *F*_1,1897_=17.97, *P*<.001). Further, virtually all participants (1962/1975, 99.3%) reported that they were continuing to play either Pokémon GO or Harry Potter: Wizards Unite during lockdown.

### Qualitative Results

#### Motivation to Play AR Games During COVID-19 Lockdown

For the first qualitative question, “What motivates you to play Pokémon GO or Harry Potter: Wizards Unite during the COVID-19 shutdown?” several key themes emerged from the participants’ responses (Multimedia Appendix 3). The most prominent themes were entertainment, achievements and challenges, in-game modifications, and exercise.

##### Entertainment

Many participants (506/1527, 33.1%) indicated that they played Pokémon GO or Harry Potter: Wizards Unite during the pandemic because these games were “fun” and “entertaining.” Some participants noted that these games provided them with “something to do” during the lockdown, as there were few alternatives they could enjoy while “stuck at home” and, in some cases, under financial constraints due to being unemployed or underemployed. Other participants mentioned that COVID-19–related restrictions gave them more time to play, with one 25-year-old male participant stating that it gave him “an opportunity to come back to the game after a few years,” that he “missed playing it,” and that it gave him “something to do in [his] downtime.”

##### Achievements and Challenges

Almost one-third of the participants (500/1527, 32.7%) stated that achievements and challenges were key motivating factors for playing Pokémon GO or Harry Potter: Wizards Unite. Participants stated that the “daily challenges” and “daily tasks” gave them a “sense of accomplishment.” Many Pokémon GO players said that they were driven to accomplish the primary objective of the game, “catch Pokémon,” as well as other objectives such as hunting for “shinies” (color-variant Pokémon) and receiving in-game rewards. Several participants specifically identified having tasks and challenges to complete in Pokémon GO and Harry Potter: Wizards Unite as providing them with beneficial temporal structure and achievable goals, given the disruptions to work or school posed by COVID-19–related restrictions. For example, one female participant aged 35 years noted that it provided “a challenge/task to complete when most of the deadlines have been removed from my life.” Another female participant aged 24 years said, “I’ve been playing Wizards Unite during the lockdown as a way to fill time and have an activity that provides a sense of progress and achievement.”

##### In-Game Modifications

Many respondents (343/1527, 22.5%) highlighted that they liked or even “loved” the new changes made to Pokémon GO and Harry Potter: Wizards Unite. The games became “more accessible to play from home,” and the “increased rewards and events have kept [people] engaged.” One 30-year-old male participant praised Niantic for their efforts: “Niantic has done well to implement features that make playing from home easier.” Notably, changes such as the “Knight Bus” in Harry Potter: Wizards Unite (a function that enabled players to cooperatively face in-game challenges from home) and the opening of the “GO Battle League” in Pokémon GO (which allowed players to challenge others and compete for rankings) reduced or eliminated the need to walk outdoors. One female Harry Potter: Wizards Unite player (age 53 years) elaborated:

The Knight Bus completely changed game play for me…Online instructions and the ability to ‘travel’ to a Fortress has made the game 100% more interesting.

Pokémon GO players also appreciated the addition of “spotlight hours, improvements to the incense and such” (male participant, age 20 years), but it was “GO Battle League” that was cited as “game-changing,” with one male player (age 30 years) mentioning that the removal of walking requirements to participate had “renewed [his] interest in the game” by providing “a measurable way to make [and measure] progress.”

##### Exercise

One-fifth of respondents (316/1527, 20.7%) noted that they used Pokémon GO or Harry Potter: Wizards Unite while exercising. Many stated that these games motivated them to exercise more, with one 24-year-old male participant mentioning that “it is the only thing that motivates me to go on walks outside” and another 33-year-old female participant saying that these games were “the only reason to step outside” and she was “actually exercising more during lockdown.” For others, it gave them “something to do during exercise” (male participant, age 29 years), or during other activities such as “walking the dog” or spending time with family members. Some elaborated on their exercise routines during the COVID-19 restrictions, with a typical example below:

Pokémon GO is the one thing that keeps me going outdoors and moving each day. It gives me a purpose to keep walking to different parts of the city but also lets me do so while staying away from clusters of other people.

Female participant, age 28 years

#### Impact of Video Games on Mental Well-being During COVID-19

Analysis of responses to the second qualitative question indicated that more than three in four participants (1102/1427, 77.2%) believed that playing video games during COVID-19 lockdown had been beneficial to their mental health; 20.7% of the participants (295/1427) gave a neutral response, and only 2.1% (30/1427) reported a negative impact. For participants who reported a positive mental health impact, several key themes described the role of video during the COVID-19 lockdown (Multimedia Appendix 4).

##### Escape/Distraction

Approximately half of the participants who reported a positive mental health impact (541/1102, 49.1%) identified that video games had been helpful in “providing a much-needed escape” and “a great distraction” from the current situation. A number of participants emphasized that games were particularly helpful in how they took their “mind off the constant depressing news coverage of COVID-19.” One 34-year-old female participant elaborated: “I can focus on the game instead of obsessively checking the news and stressing out.” Another male participant (age 27 years) said: “[They] kept me away from watching the news all day. It was quite nice to not be surrounded by COVID news all the time.”

##### Activity/Entertainment

Almost half of the participants (535/1102, 48.5%) stated that video games gave them “something to do,” helped them to “stay busy,” and kept them “entertained” during COVID-19 lockdown. Games gave them “something fun to focus on” and “helped to relieve boredom” while “being at home 24/7.” Many described how games gave them “something to look forward to,” with one 30-year-old male participant even stating that beyond keeping him “motivated and busy,” games helped him to “get out of bed and do something.”

##### Emotional Coping

In addition to providing an escape and serving as a way to pass the time or be entertained, many participants (469/1102, 42.6%) identified that video games had helped them “cope” and maintain “a calm and positive outlook.” Playing video games allowed them to “relieve some stress” and “relax,” and it “lifted [their] mood.” One 30-year-old female participant wrote that video games were “possibly the only thing keeping me sane right now.” Some respondents also wrote about using video games to alleviate specific mental health conditions:

It’s kept me in a safe place mentally. While college was in session I had depression and anxiety and it’s still lingering as of now. It takes my mind off of the outside world.

Male participant, age 19 years

I suffered from anxiety and depression prior to the shutdown, too, and used games for the same escape then.

Female participant, age 42 years

##### Social Connection

One in five respondents (219/1102, 19.9%) emphasized the importance of video games in strengthening social connections. Many indicated that they played video games with others in their household (eg, children and partner), thus “providing bonding opportunities.” Multiplayer games provided an opportunity to stay in touch with friends and “still share in something together.” One 30-year-old participant stated: “It has helped because it’s the only social interaction I get.” Some participants highlighted the importance of the local “community” these games create, noting that playing let them feel “less isolated”:

In Pokémon GO, the local app group makes me feel part of a community, even if we cannot all meet face to face right now.”

Female participant, age 31 years

My HWPU [Harry Potter: Wizards Unite]/ingress community connections have been vital in having someone to talk to, and learning about other people's experiences.

Female participant, aged 54 years

## Discussion

### Principal Results

The COVID-19 pandemic has significantly disrupted normal activities worldwide. In this study, we sought to examine the impact of COVID-19 on people’s physical and mental well-being and on video game use. We also sought to explore the potential role of video games (and AR games in particular) in supporting the mental well-being of players during COVID-19 lockdown. We summarize our key findings in accordance with our three objectives.

#### 1. Impact of COVID-19–Related Social Restrictions on the Physical and Mental Well-being of AR Game Players

Due to the COVID-19–related stay-at-home restrictions, exercise levels dropped significantly from an average of 7.5 hours to 6.5 hours per week during the pandemic. Despite this drop, on average, players continued to exercise well above the level of 150 minutes (2.5 hours) of moderately intense activity recommended per week by the WHO to achieve health benefits [30]. Previous research has identified that performing at least 150 minutes of exercise a week improves cardiorespiratory and muscular fitness, improves bone health, and reduces the risk of chronic disease and depression [30]. From the quantitative survey data, we also identified a significant positive relationship between total hours of participation in gaming and total hours of exercise per week, indicating that rather than hardening unhealthy lifestyle patterns [6], AR games may be continuing to promote physical activity, even with the restrictions due to COVID-19. This finding was also supported by our qualitative results, with players consistently mentioning that AR games were keeping them motivated to exercise during the pandemic. This is particularly important because our results also show that reduced exercise is a significant predictor of poor mental health, even after taking participants’ gender and age into account.

Further, consistent with emerging research [31], our study suggests that the impact of COVID-19 lockdown on mental well-being is high. Over half of participants in this study (925/1766, 52.4%) reported poor mental health. Compared to the previous estimated prevalence of poor mental health of around 25% [32], our identified prevalence of over 50% suggests that these numbers have doubled during the COVID-19 pandemic. A similar substantial impact of the current pandemic situation on mental health has been identified in other recent studies [31,33]. For example, research on large nationally representative samples of adults in the United States reported that depression symptom prevalence has more than doubled during the COVID-19 pandemic, with 52.5% of the population showing symptoms of mild depression or greater, compared to 24.7% before the pandemic [34]. The results from this emerging research on the mental health impact of COVID-19 are considerable cause for concern.

#### 2. Impact of COVID-19–Related Social Restrictions on Motivation to Play and Video Game Use Among AR Game Players

Consistent with reports from popular media, this study found a significant increase in the use of video games during COVID-19 lockdown, mostly driven by casual gamers—women in particular—increasing their video game play. Despite the inconveniences to normal gameplay caused by COVID-19 restrictions, nearly all respondents continued to play Pokémon GO and Harry Potter: Wizards Unite. In fact, the qualitative results indicated that the in-game changes Niantic made to Pokémon GO and Harry Potter: Wizards Unite were a key motivating factor in players’ continued use of the game during the pandemic, with many reporting that they “loved” the changes or had returned to the game because of them. This is also consistent with recent research on social media reactions across three popular Pokémon GO subreddits, which showed overwhelming appreciation toward the in-game changes to Pokémon GO [10].

#### 3. Potential Role of AR Games in Supporting the Mental Well-being of Players During COVID-19–Related Social Restrictions

Despite previous concerns about increased video game use during COVID-19 lockdown [6], more than three in four participants in this study suggested that video games helped support their mental health. The qualitative results suggest that during ongoing lockdown and social distancing measures, video games provided an escape from the fear accompanying the pandemic and provided players with something to do while their usual routines (eg, work, school, hobbies) were disrupted. Beyond that, video games were also being used to aid emotional coping—to lower stress, relax, and alleviate specific mental health conditions. Respondents also reported that games played a critical role in maintaining social connections and encouraging physical activity. Recent research has highlighted the importance of social connection [35,36] and exercise [37] to mitigate the negative psychological consequences of the COVID-19 pandemic; thus, these games represent a beneficial coping mechanism for their players.

### Limitations

Our study was limited in ways that must be taken into consideration when interpreting the results. First, although the study drew participants from 66 countries, the survey was conducted in the English language only, thus excluding participation by non-English speakers. Examining cultural effects on AR game use and gamers’ well-being could be an interesting area for future research. Second, our survey was cross-sectional; therefore, we are unable to imply causality between game play and physical activity based on the current study alone. However, we have examined the data in ways that provide clear direction for future longitudinal research. Third, our study may be subject to sample bias, with recruitment occurring via four subreddit forums dedicated to Pokémon GO or Harry Potter: Wizards Unite; thus, the results may not be generalizable to the entire population. Fourth, this study also relied on self-reported data, which creates potential for recall bias. For our mental health assessment, we relied on the WHO-5 as a noninvasive assessment of subjective well-being. Although this measure cannot be used to clinically diagnose mental illness, previous research supports the use of the WHO-5 to effectively screen for depression. However, further research would benefit from the inclusion of more specific mental health issues, such as the Depression Anxiety Stress Scales [38] and the UCLA Loneliness Scale, to assess social isolation [39]. Women are also known to be less likely to self-identify as people who play video games than men [23,40,41]; this may have contributed to under-reporting of the number of participants who were “hardcore gamers,” especially for female respondents. Finally, the international scope of our sample also means that different participants had different COVID-19 lockdown experiences, as some countries enforced strict lockdowns at the time of the survey while others imposed few social limitations [42].

### Comparison With Prior Work

Prior to the COVID-19 pandemic, the potential of video games to improve people’s physical and mental well-being [15] had already been well demonstrated. Beyond providing entertainment, games have the capacity to foster positive emotions [43,44], create engagement and commitment [45,46], encourage new and long-lasting social relationships [47], provide purpose and meaning to daily life [48], and deliver a sense of accomplishment and competence [46,49]. Exergames, defined as video games that mediate physical exercise [50], have been reported to be effective in clinical rehabilitation [51,52] and general exercise promotion [53,54]. They also provide holistic, cross-sectional improvements in mental health [55,56]. Within the first six months of the release of Pokémon GO, the game had already been recognized as an extremely complex behavioral intervention [57,58] that increased the duration of physical exercise for all its players, regardless of socioeconomic status [13]. Individuals and organizations worldwide also started to use games during the COVID-19 shutdown as a means of health education, mental coping, social bonding, and providing a sense of control and routine [59]. Our findings, which indicated that Pokémon GO and Harry Potter: Wizards Unite players are supporting players’ physical and mental well-being, support much of this earlier work.

A recent report from Canada [60] stated that adults are spending more time on “screens” during the pandemic. The authors of this study argued that an increase in sedentary screen time may be contributing to a decline in mental well-being during COVID-19 lockdown. In line with this argument, we found that reduced exercise was a significant predictor of poor mental health; this finding is consistent with previous pre–COVID-19 research correlating increased exercise with positive mental health [61-63]. However, the focus of the Canadian study was not on AR games such as Pokémon GO and Harry Potter: Wizards Unite. Our study suggested that playing more Pokémon GO and Harry Potter: Wizards Unite maintained participants’ weekly exercise well above the recommended levels of 150 minutes of moderate exercise per week [64] and helped preserve players’ mental well-being. Specifically, our study indicated that Pokémon GO and Harry Potter: Wizards Unite motivated participants to maintain a walking and socialization schedule. These behaviors reduced loneliness, which is known to correlate with worse mental health during the COVID-19 shutdown [35,65,66]. Interestingly, our qualitative findings identified that casual players, who previously played Pokémon GO and Harry Potter: Wizards Unite less frequently than the hardcore players, are now playing as much as members of the hardcore group. Further, the qualitative findings suggest that casual players, who are usually less engaged with games, are using games to both distract themselves from the pandemic and provide themselves with a sense of challenge and achievement that they usually source from activities that are currently disrupted by the pandemic.

Participants in this study indicated that they were using games in general to stabilize their mood and prevent further emotional stress. Overwhelmingly, the participants indicated that video games were beneficial to their mental health. Holistically, AR games such as Pokémon GO and Harry Potter: Wizards Unite provide virtual socialization with other people, motivate players to sustain exercise, and offer a daily routine during the lockdown, therefore establishing mental coping strategies to benefit well-being during COVID-19 lockdown. As stated recently in a commentary on gaming in the time of COVID-19, Kriz [67] suggested that “games have the potential to entertain and relax, to lower stress (maybe even provide an escape for a while from the real problems and fear accompanying the pandemic) and to provide the opportunity to interact with friends through (virtual) forms of play.”

### Conclusions

Location-based AR games such as Pokémon GO and Harry Potter: Wizards Unite already held the potential to be deployed as digital interventions with a beneficial impact on physical and mental health before the COVID-19 pandemic. We provide insight into the effects of continued playing of Pokémon GO or Harry Potter: Wizards Unite on a global sample of AR players. Participants reported increased game play, which coincided with their maintaining exercise levels well above international recommended levels, and using these games as a means of mental coping. Increased game play was significantly associated with increased exercise and was not associated with worse mental health during COVID-19 lockdown.

Mentally, our participants reported they continued to play the games out of a desire for entertainment, to have some structure in their daily lives, to have a feeling of accomplishment, and as a means to continue exercising. On a larger scale, the participants played video games to cope with the negative psychological impact of COVID-19 lockdown. They used games to distract themselves, escape briefly from reality, have something to do during their days, manage their emotions and mental health, and stay socially connected with other people. User goals such as these indicate that Pokémon GO and Harry Potter: Wizards Unite were already being used as veritable digital behavioral interventions by the participants to self-regulate emotional distress, especially during the periods of intensive social isolation associated with the COVID-19 pandemic.

By understanding the phenomenon of how game players worldwide are using the interactive and immersive medium of video games to manage their own mental health, we are glimpsing the massive potential video games have to become behavioral “medicines” for populations under stress. Location-based AR games have greater potential due to their propensity to encourage exercise and social connections. It would be beneficial for the health care system and digital health professionals to leverage this interactive software effectively during the mental health crisis emerging during COVID-19 lockdown, providing virtual relief and maintaining holistic well-being.

## Appendix

Multimedia Appendix 1 Questions asked in the web-based survey presented in this study.

Multimedia Appendix 2 Multivariate binary logistic regression analysis.

Multimedia Appendix 3 Summary themes identified for motivation to play Pokémon GO or Harry Potter: Wizards Unite (N=1527).

Multimedia Appendix 4 Summary themes identified for the impact of video games on mental health (N=1427).

## Abbreviations

ANOVA: analysis of variance
AR: augmented reality
WHO: World Health Organization
WHO-5: World Health Organization–5 Well-Being Index
