# Supplementary material for: COVID-19 as ‘Game Changer’ for the Physical Activity and Mental Well-Being of Augmented Reality Game Players During the Pandemic: Mixed Methods Survey Study
Source: J Med Internet Res. 2020 Dec 22;22(12):e25117. doi: 10.2196/25117 (PMC7758086; doi:10.2196/25117)
Supplement: Multimedia Appendix 1 [file jmir_v22i12e25117_app1.pdf]

Multimedia Appendix 1 – Online survey questions presented in this study

**Qu #**	**Area/sub area**	**Question text**	**Answer options/format**
1	Demographics	What is your gender?	*Select one:*<br>- Male<br>- Female<br>- Other<br>- Prefer not to answer
2	Demographics	What is your age?	Numerical entry box
3	Demographics	What is your country of residence?	Country list dropdown box from Qualtrics
4	Self-identified player type	Regarding video games, I would consider myself a:	*Select one*<br>- Hard core gamer<br>- Casual gamer<br>- Something in between<br>- I have no idea<br>- Prefers not to say
10	Video game use	Prior to the COVID-19 shutdown, how many days a week were you playing either *Pokémon GO* or *Wizards Unite*?	Numerical entry box

Qu #	Area/sub area	Question text	Answer options/format
11	Video game use	Prior to the COVID-19 shutdown, how many hours a day were you playing either *Pokémon GO* or *Wizards Unite* during the days that you did play?	Numerical entry box
12	Video game use	Are you still playing either *Pokémon GO* or *Wizards Unite* during the shutdown*?*	*Select one*<br>- Yes<br>- No
13	Video game use	How many days a week do you play now?	*[Noted only presented if 'yes' was selected for Q12]*<br>Numerical entry box
14	Video game use	How many hours a day do you play now, during the days that you do play?	*[Noted only presented if 'yes' was selected for Q12]*<br>Numerical entry box
15	Exercise	Prior to the COVID-19 shutdown, how many days a week were you exercising?	Numerical entry box
16	Exercise	Prior to the COVID-19 shutdown, how many hours a day were you	Numerical entry box

Qu #	Area/sub area	Question text	Answer options/format
		exercising during the days that you did physical exercises?	
17	Exercise	How many days a week do you exercise <u>now</u>?	Numerical entry box
18	Exercise	How many hours a day do you exercise <u>now</u>, during the days that you do physical exercises?	Numerical entry box
20	Mental well-being (WHO-5)	During the past 2 weeks...<br><br>- I have felt cheerful and in good spirits<br>- I have felt calm and relaxed<br>- I have felt active and vigorous<br>- I woke up feeling fresh and rested<br>- My daily life has been filled with things that interest me	*Likert scale- select one per item*<br><br>- All of the time<br>- Most of the time<br>- More than half the time<br>- Less than half the time<br>- Some of the time<br>- At no time
21	Motivation to play	What motivates you to playing *Pokémon GO* or *Wizards Unite* during the COVID-19 shutdown?	Open-ended text box

Qu #	Area/sub area	Question text	Answer options/format
29	Mental well-being	How has playing video games affected your mental health during the shutdown?	Open-ended text box
