# Supplementary material for: COVID-19 as ‘Game Changer’ for the Physical Activity and Mental Well-Being of Augmented Reality Game Players During the Pandemic: Mixed Methods Survey Study
Source: J Med Internet Res. 2020 Dec 22;22(12):e25117. doi: 10.2196/25117 (PMC7758086; doi:10.2196/25117)
Supplement: Multimedia Appendix 2 [file jmir_v22i12e25117_app2.pdf]

Multimedia Appendix 2 – Multivariate binary logistic regression analysis[a]

Variable	B	SE	Wald's χ^2	df	*P*	OR	OR CI 95%
Constant	.397	.206	3.711	1	.054		
Gender	.672	.115	33.923	1	.000**	1.958	1.562 –2.455
Age	-.020	.006	11.148	1	.001*	0.980	0.969 – 0.992
Self-identified player type			3.074	2	.215		
HvCM	-.174	.155	1.270	1	.260	0.840	0.621 – 1.137
CvHM	.890	.123	.530	1	.467	1.093	0.860 – 1.390
Game Play	-.007	.003	4.245	1	.39	0.993	0.987 – 1.000
Exercise	-.016	.006	6.991	1	.008*	0.984	0.972 – 0.996

Notes: [a]R2 =0.040 (Cox and Snell); 0.054 (Nagelkerke); Model χ^2 (6, N=1469)= 60.538; ****P***<.01 (two-tailed), ** ***P*** <.001 (two-tailed); Gender (Male=0, Female=1); HvCM (Hardcode=1, Casual=0, Midcore=0); CvHM (Casual=1, Hardcore=0, Midcore=0); Game play= Game play change score (during-COVID minus pre-COVID); Exercise=Exercise change score (during-COVID minus pre-COVID).
Abbreviations: SE, standard error; df, degrees of freedom; OR, odds ratio; CI, confidence interval.
