# Supplementary material for: COVID-19 as ‘Game Changer’ for the Physical Activity and Mental Well-Being of Augmented Reality Game Players During the Pandemic: Mixed Methods Survey Study
Source: J Med Internet Res. 2020 Dec 22;22(12):e25117. doi: 10.2196/25117 (PMC7758086; doi:10.2196/25117)
Supplement: Multimedia Appendix 3 [file jmir_v22i12e25117_app3.pdf]

Multimedia Appendix 3: Summary themes identified for motivation to play PGO or HPWU (N=1527)

**Theme**	**Representative quotes**	**n (%)**
1. Entertainment	"Pokémon go is just fun"<br>"Playing these games gives me something to do with the time I would not normally have"<br>"I really enjoy the game and have plenty of free time on my hands"<br>"Something to do instead of being stuck at home"<br>"It's a fun way to pass the time. I love Harry Potter"<br>"I missed playing it, and this gave me an opportunity to come back to the game after a few years. It gives me something to do in my downtime and gets me moving a bit more"	506 (33.1)
2. Achievement/Challenge	"Completing quests"<br>"Daily challenges, sense of accomplishment"<br>"It provides some sort of daily goal to accomplish while my workplace is temporarily frozen"<br>"Gotta Catch 'Em All"<br>"To keep my daily streak going and to try and grind to level 40"<br>"It gives me a daily goal during this pandemic"	500 (32.7)
3. In-game modifications	"Enhanced play from home functionality, especially the addition of the Wizards Unite Knight Bus. Fortresses have always been one of my favourite parts of the game, but as a player in a fairly rural community I had never been able to	343 (22.5)

	participate in any grouping bigger than a pair with my SO. I can now fortress with my WU friends from around the world, a feature I had always hoped for before the shutdown"<br>"The Knight Bus completely changed game play for me. I have no local fortresses and didn't have time/inclination to go park by one to actually do the challenges. I also don't know anyone near me who plays so I had no team members to learn with. Online instructions and the ability to "travel" to a Fortress has made the game 100% more interesting"<br>"I feel like Niantic has made changes (because of corona) that have benefit Pokemon Go and made it more fun to play if you are at home, like the spotlight hours, improvements to the incense and such. So overall it is still a lot of fun to play the game"<br>"My interest in Go actually increased quite a bit during covid due to removing the battle league walking requirements. I mostly play Go for battle league these days, and I hope the walking requirement isn't reinstated"<br>"I already have quite a bit of motivation to walk to earn candies so I can build up new Pokémon for battle league"	
4. Exercise	"It motivates me to go out for walks"<br>"PG and HPWU are the only reasons I go for a walk nowadays"<br>"It is the only thing that motivates me to go on walks outside"	316 (20.7)

	"I am overweight and it keeps me on walks longer. It gives me something to do while exercising that I normally wouldn't have to do" "I picked the game up again during the shutdown because I'm going on many more walks for fun, and it seemed like a good way to keep encouraging me to go on walks"	
5. Routine	"They've become an important part of my daily routine since this shut-down" "Part of my daily routine" "It's been a daily habit for a long time"	257 (16.8)
6. Social connection	"I feel a bit less isolated playing" "To spend time with family (who play and live in the same household)" "Spend time with friends online" "I have discovered a group to play with online" "Talking with other players on Discord, FB Messenger, etc about current events within the games"	173 (11.3)
