# Supplementary material for: COVID-19 as ‘Game Changer’ for the Physical Activity and Mental Well-Being of Augmented Reality Game Players During the Pandemic: Mixed Methods Survey Study
Source: J Med Internet Res. 2020 Dec 22;22(12):e25117. doi: 10.2196/25117 (PMC7758086; doi:10.2196/25117)
Supplement: Multimedia Appendix 4 [file jmir_v22i12e25117_app4.pdf]

Multimedia Appendix 4: Summary themes identified for impact of video games on mental health (N=1427)

**Theme**	**Representative quotes**	**n (%)**
Positive benefit		1102 (77.2)
1. Escape/distraction	"Something to distract from all the bad stuff happening around us during this time"<br>"It's a way to escape the horrors of the real world and just have a fun time in a world where you can be whatever you want to be"<br>"It's kept my mind off the news"<br>"It has highly increased my mental health, making me forget about Covid-19"<br>"Allows for a healthy escape to another world"	541 (49.1)
2. Activity/entertainment	"I think it's given me something to do so that I don't go crazy staying at home all the time"<br>"WU and Pokémon Go give me something to look forward to"<br>"Gave me something to do. Avoiding boredom"<br>"It's a nice way to pass the time…"<br>"They keep me entertained and my mind busy"	535 (48.5)
3. Emotional coping	"Possibly the only thing keeping me sane right now"<br>"Helped keep me calm and level headed and not to over worry about what is going on in the state of the world."<br>"It helps with my anxiety"	469 (42.6)

	"Usually lifts my mood even if it's just by a bit"<br>"Helps with my stress and anger from the current living situation"<br>"They also bring a great deal of joy to my life which is otherwise very bleak at the moment"<br>"Helped keep me stable"<br>"It helps to get me out on a walk even when I don't feel motivated, and the walk helps manage my stress and anxiety"	
4. Social connection	"It keeps me sane. I do a lot for my community and everyone is happy to see me. Everyone trusts me to help them"<br>"It has helped because it's the only social interaction I get"<br>"It's been helpful; been staying in touch with friends and having fun"<br>"I am playing with my friends a lot during this shutdown. I find it relaxing and great to keep in contact during these times"<br>"Having something my whole family can happily do together has been very helpful"<br>"I'm glad they exist, and I'm glad for the sense of community some of them create"<br>"My HWPU/ingress community connections have been vital in having someone to talk to, and learning about other people's experiences"	219 (19.9)

5. Achievement/Challenge	“It has helped me positively and has kept me focusing on small wins in the game. It has definitely become an outlet for my health”<br>“Helps immensely to keep the mind active and achieve goals/progress on things”<br>“It has given me little goals to work towards so I can still feel like I am achieving things even though they're only for a game”	173 (15.7)
6. Routine	“It’s my rock of normality”<br>“Normality through routines before lockdown”<br>It helped relax and structure the day”<br>“Provides a lot of motivation and structure to my day that helps everyday not feel the same”<br>“Playing video games at certain times have offered structure through my day, helping it feel more 'normal'”<br>“I enjoy Pokemon Go and I feel like I can continue to work towards goals and do something I was doing before still. So it is some continuity in life, which there isn't a lot of otherwise!”	137 (12.5)
Neutral impact	“Neutral impact”<br>“No effect”<br>“No change”	295 (20.7%)
Negative impact	“Addicted”<br>“Playing too much” is “bad”	30 (2.0%)

	Feel bad about “wasting time” playing these games and wished that they spent the time “on something productive despite the shutdown”	
